# The Relationship between Eosinophil Density in the Colonic Mucosa and Eosinophil Blood Count in Children: A Cross-Sectional Study

**DOI:** 10.3390/children10010006

**Published:** 2022-12-21

**Authors:** Jan Brylak, Jan K. Nowak, Mariusz Szczepanik, Magdalena Holubiec, Pawel Kurzawa, Jaroslaw Walkowiak

**Affiliations:** 1 Department of Pediatric Gastroenterology and Metabolic Diseases, Poznan University of Medical Sciences, Szpitalna 27/33, 60-572 Poznan, Poland; 2 Department of Oncological Pathology, Heliodor Swiecicki Clinical Hospital in Poznan, Poznan University of Medical Sciences, Przybyszewskiego 49, 60-355 Poznan, Poland; 3 Department of Clinical Pathology and Immunology, Poznan University of Medical Sciences, Przybyszewskiego 49, 60-355 Poznan, Poland

**Keywords:** eosinophil, Crohn’s disease, ulcerative colitis, colonoscopy, mucosal density, histopathology, IgE

## Abstract

Eosinophils are found in the mucosa of the healthy gastrointestinal tract, but they also often accompany gastrointestinal diseases. We hypothesized that a positive correlation exists between blood eosinophil count and colonic eosinophil mucosal density in children. Electronic health records regarding 181 colonoscopies, performed with biopsy in the years 2019–2022, were screened for information on blood and colonic eosinophil count, age, sex, diagnoses, weight, height, white blood cell (WBC) count, serum C-reactive protein (CRP), and total IgE concentration. The median age (IQR) of the 107 included children (109 colonoscopies) was 12.4 years (8.1–15.5); 32 presented with blood eosinophilia (29.3%). The median eosinophil density/high-power field in the colonic mucosa was 22.5 (9–31). We found a weak correlation between colonic mucosal eosinophil density and blood eosinophil count (r = 0.295, 95% CI 0.108–0.462, *p* = 0.0018). This association was more pronounced in patients with elevated CRP (r = 0.529, 95% CI 0.167–0.766, *p* = 0.0054) and older than 12.4 years (r = 0.448, 95% CI 0.197–0.644, *p* = 0.00068). Peripheral blood eosinophilia might hint at increased mucosal colonic eosinophil density, especially in older children and in the presence of systemic inflammation. However, it seems unlikely that blood and colonic eosinophilia are strongly linked in younger children. Studies in adults are warranted.

## 1. Introduction

Eosinophils are a subtype of white blood cells (WBCs) found in the immune system in humans and all other vertebrates [1]. They normally constitute between 1 and 3–5% of circulating WBCs in humans [1,2], where they play a crucial role in the reaction to parasites and allergens. It is proposed that eosinophils also facilitate tissue remodeling and coordinate immune regulation, but further investigation in these areas is still needed [3]. In healthy people, eosinophils are frequently found in the mucosa of the gastrointestinal tract [4], with the exception of the esophagus [3].

The increased number of eosinophils in the bloodstream may be related to a vast number of underlying conditions including allergic diseases and asthma, parasitic infections and medication intake (e.g., penicillins, cephalosporins, NSAIDs) [5,6,7]. The eosinophilic infiltration related to most of these causes may also involve the gastrointestinal tract. Eosinophilic esophagitis (EoE), eosinophilic gastroenteritis and eosinophilic colitis are thought to be specific eosinophil-driven diseases of the gut [8,9,10]. Eosinophilia in the peripheral blood is often listed among the criteria for diagnosing eosinophilic maladies of the gastrointestinal tract [11].

In children with severe asthma, blood and airway eosinophilia were correlated, but the negative predictive value of normal blood eosinophil count was insufficient [12]. Nevertheless, Bedolla-Barajas et al. indicated a positive correlation between asthma severity and blood eosinophilia [13]. Similarly, Zeiger et al. showed a positive link between blood eosinophilia and asthma exacerbation risk [14]. In EoE blood, eosinophilic count significantly correlates with esophagus mucosal eosinophil density [15,16]. Furthermore, eosinophil count is sometimes considered a potentially useful parameter in the assessment of treatment effectiveness, which often requires repeated gastroscopy studies [17,18]. In pediatric ulcerative colitis, colonic or blood eosinophilia was associated with inflammation severity, but was not a long-term prognostication marker [19,20]. Yet, in adults with ulcerative colitis, recurrent eosinophilia was associated with severe colitis and primary sclerosing cholangitis [21]. Whether eosinophilic count is useful for monitoring treatment efficacy in eosinophilic colitis requires further research; the current evidence on this topic is negative [22].

Very little is known about the correlation between the concentration of eosinophils in the bloodstream and the gut. Therefore, we hypothesized that eosinophil count in the blood positively correlates with the mean eosinophil density found in the biopsies of colonic mucosa across a group of children undergoing endoscopy.

## 2. Materials and Methods

This was a retrospective cross-sectional study. Records of 181 patients who underwent colonoscopy with colon biopsy in the years 2019–2022 were screened for information on both blood eosinophil counts and the presence of eosinophils on histopathological examination. From the documentation, we obtained information on age, sex, diagnoses, weight, height, total white blood cells (WBC) count, serum C-reactive protein (CRP), and total IgE concentrations. The blood count was analyzed with automatic measurement methods using a Sysmex 5-differential hematology analyzer in the hospital laboratory. Information on the eosinophil density (per one high-power field, HPF) was extracted from histopathology reports and, if only descriptive terms were available, they were interpreted according to typical practice in our laboratory. Over 90% of biopsy reports were prepared by the same pathologist and all of them came from the same center. Data analysis focused on correlations in the whole group and specific subgroups. The data were analyzed using PQStat 1.8.4.138 (PQStat Software, Poznan, Poland). Non-parametric statistics are presented for the investigated parameters. Spearman’s correlation coefficient was calculated to investigate monotonic relationships (Spearman’s rho (ρ) is presented as r). A multi-variable regression model was built. The significance threshold was set at 0.05.

## 3. Results

The search identified 109 subjects for whom the information on blood and tissue eosinophils was available. Patients had typical indications for colonoscopy, including hematochezia, diarrhea and abdominal pain. The most common final diagnoses were ulcerative colitis (*n* = 34, 31.2%) and Crohn’s disease (*n* = 16, 14.7%). Eight patients (7.3%) had mild proctitis that did not progress to ulcerative colitis. One patient with familial adenomatous polyposis was included, and one with a juvenile polyp, because biopsies of normal mucosa were available (1.8%). The study did not reveal significant colonic pathology in the remaining patients (*n* = 49, 44.9%), among whom there were cases of gastritis, duodenitis, infection with *Helicobacter pylori* (the urease test) and *Yersina enterocolitica* (positive serology), and hemorrhoids.

The median age (1st–3rd quartile) was 12.4 years (8.1–15.5), and the age range was 1–18 years (Table 1). Fifty-two study participants were female (47.7%). The median weight and height were 42 kg (25–58) and 152 cm (130–165). Most of the subjects did not have elevated CRP and the median was 0.04 mg/dL (0–0.45), with 26 presenting elevated values (23.8%). However, in almost all cases, CRP was less than ten times over the upper limit of the norm (which was 0.5 mg/dL). The median WBC concentration was 7.64 G/L (5.9–9.1).

**Figure 1:**
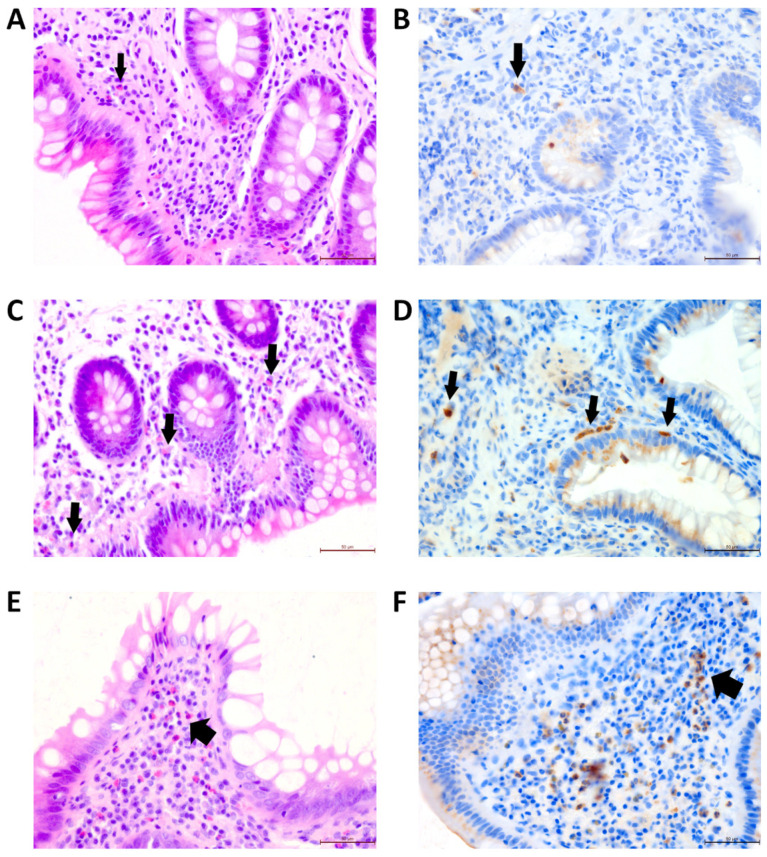
(**A**) Fragment of the mucosa of the large intestine with sparse intestinal eosinophils (arrows →), H&E, mag. 400×; (**B**) stained positive for myeloperoxidase (MPO). (**C**) Fragment of large intestine mucosa with 20–25 intestinal eosinophils/1 HPF (H&E); (**D**) stained positive for MPO. (**E**) Fragment of colonic mucosa with over 40 intestinal eosinophils/1 HPF (H&E); (**F**) stained positive for MPO. Bar length in all panels: 50 µm.

The median eosinophil count was 0.16 G/L (0.10–0.31), with 74 results within the norm (67.9%), 32 above (29.3%) and 3 below (2.7%). Relative to the total WBC count, the median fraction of eosinophils was 2.5% (1.4–3.7%). The IgE level was available for 76 patients (69.7%) and the median was 44.3 kU/L (13.1–161.5), with seven subjects presenting elevated values [23]. In histopathological analysis, the eosinophils were assessed on average 2.3 times per patient. The median eosinophil density/HPF in the colonic mucosa was 22.5/HPF [9,10,11,12,13,14,15,16,17,18,19,20,21,22,23,24,25,26,27,28,29,30,31] (Figure 1). The girls and the boys did not differ significantly in median colonic eosinophil density (*p* = 0.193), eosinophil count in the blood (*p* = 0.337) or in any of the other investigated parameters (all *p* > 0.4).

**Figure 2:**
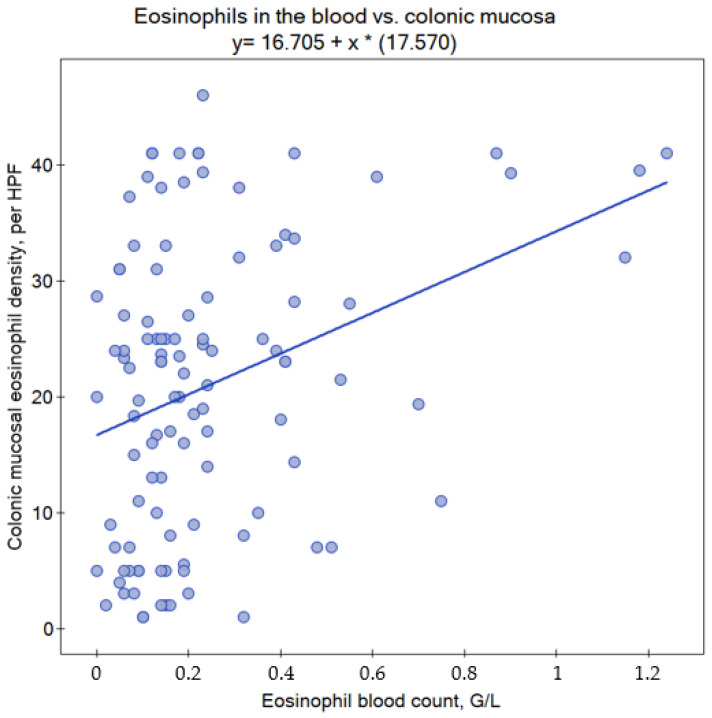
Scatterplot of colonic mucosal eosinophil density vs. eosinophil blood count in 109 children who underwent diagnostic colonoscopy. Some points may overlap. HPF—high-power field. Asterisk (*) indicates multiplication.

The colonic mucosal eosinophil density weakly correlated with blood eosinophil count (r = 0.295, 95% CI 0.108–0.462, *p* = 0.0018; Figure 2), but not with eosinophil count as a percentage of WBCs (r = 0.175, 95% CI −0.019–0.356, *p* = 0.069). However, there was an association between mucosal eosinophil density and WBC count (r = 0.262, 95% CI 0.072–0.433, *p* = 0.0059). The relationship between colonic eosinophil density and CRP did not reach statistical significance (r = 0.182, 95% CI −0.013–0.364, *p* = 0.060). There was also no correlation between colonic mucosal eosinophil density and total IgE levels (r = −0.024, 95% CI −0.254–0.208, *p* = 0.833) (Table 2). None of these relationships were statistically significant when only children below the median age were considered (<12.4 years), but interpretation needs to take into account the fact that, in this subgroup, *p* values were <0.3 (and the sample size was two times smaller). However, in older subjects (>12.4 years), despite the smaller sample size, the relationship between mucosal eosinophil density and eosinophil blood count was moderately strong (r = 0.448, 95% CI 0.197–0.644, *p* = 0.000676), and there was also a positive association with relative eosinophil frequency in the blood (r = 0.432, 95% CI 0.177–0.632, *p* = 0.0011) and with total WBC count (r = 0.300, 95% CI 0.026–0.531, *p* = 0.027). The correlation, seen between mucosal eosinophil density and blood eosinophil count, was preserved in subjects (all ages) with normal (r = 0.245, 95% CI 0.022–0.445, *p* = 0.027) and elevated CRP alike (r = 0.529, 95% CI 0.167–0.766, *p* = 0.0053). Finally, in children who were diagnosed with ulcerative colitis, a correlation between eosinophil count and mucosal eosinophil density was detected (r = 0.530, *p* = 0.0013). We did not find any statistically significant correlations in a subgroup of 27 subjects who had total IgE > 100 kU/L (*n* = 27). Regression analysis revealed that among age, gender, CRP, WBC and eosinophil count, only the latter was independently associated with colonic eosinophil density (model R^2^ = 0.161, *p* = 0.0028). The beta coefficient for the eosinophil count was 16.07 (95% CI 6.02–26.12, *p* = 0.0020). The result was significant also after inclusion of the final diagnosis category in the model (ulcerative colitis, Crohn’s disease, other).

Higher colonic mucosal eosinophil density was found in ulcerative colitis (32.5 (25.0–39.0) per HPF), compared with both Crohn’s disease (19.5 (10.0–24.5), *p* = 0.0021) and the remaining diagnoses (18 (7–24), *p* = 0.000002). Total IgE concentrations were slightly lower in ulcerative colitis compared with Crohn’s disease (22.9 (12.1–101.0) vs. 81.3 (49.3–383.0) kU/L, *p* = 0.037). Subjects with inflammatory bowel diseases did not differ in age, mass or height from other patients.

## 4. Discussion

In the present study, we found a correlation between colonic mucosal eosinophil density and blood eosinophil count. The association was more pronounced in patients with elevated CRP and adolescents older than 12.4 years (i.e., the median age). In contrast, the association was not confirmed in younger children. We also found a modest correlation between colonic mucosal eosinophil density and WBC count, both in the studied population as a whole and among patients > 12.4 years. Surprisingly, we found no association between total serum IgE and colonic mucosal eosinophil density. The subject is understudied and there are only a few studies to compare independent findings with our analysis.

### 4.1. Molecular Aspects of Eosinophilia

Our knowledge of the eosinophil is mostly related to the airway mucosa because of the importance of this type of cell in asthma. In gastroenterology, more recent research has focused mostly on EoE and not on physiological functions of the eosinophil in the intestine. The pathophysiology of eosinophilia beyond the esophagus relates to allergy and dysbiosis, both of which are briefly discussed below, together with a general overview of the mucosal eosinophil [24].

The eosinophil originates from the granulocyte lineage and does not multiply. It is found where the organism meets the environment: in the gut, lungs, skin, and also in the lymph nodes and spleen. The eosinophil is able to survive in tissues longer than other granulocytes. Activation of the eosinophil may lead to its rupture, with the spread of granule content, which contains a mixture of effector proteins, cytokines and leukotrienes [25]. The key protein for anti-helminthic, antibacterial and cytotoxic effects is the major basic protein, which also stimulates the release of histamine. Other eosinophilic granule proteins include enzymes that may have direct activity on the offending organisms or help to produce reactive oxygen species. Eosinophils respond to various stimuli, one of which is IgE. However, IgE alone is much more likely to promote degranulation of mastocytes, and not eosinophils. Pathways involving IgE and eosinophils (IL-5) are considered as distinct in the context of targeted therapies [26].

Eosinophils are recruited by such inflammatory factors as IL-5, TNF and IL-1beta, but also the hypoxia-inducible factors. The strongest eosinophil chemoattractant in the mucosa is eotaxin-1, secreted by healthy intestinal lamina propria. Mucosal eosinophils acquire markers of activation, including ICAM-1 and CD69, remaining in a state of alertness, ready to react to bacterial products. Recent research implicated the aryl hydrocarbon receptor as a key factor in promoting the optimal level of eosinophil reactivity in intestinal mucosa [27] that prevents premature degranulation. Importantly, eosinophils are able to present antigens via the major histocompatibility complex (MHC) class 2. Eosinophils are therefore involved in maintaining mucosal integrity and are closely linked with other immune cells. The view of the eosinophil as an effector cell and actor of inflammation should be reconciled with its potential functions in the early priming of inflammation (e.g., via the aforementioned antigen presenting capacities). Moreover, eosinophils are also able to secrete anti-inflammatory proteins (IL-10 and TGF-beta), influence tissue healing and promote fibrosis. It is often difficult to distinguish whether eosinophils in the biopsy are pro-inflammatory or take part in repair [28]. Furthermore, even the inflammatory activity of eosinophils may be difficult to categorize as primary (EoE) or secondary (gastroesophageal reflux disease) without a broad knowledge of the preexisting pathology, coupled with information about past and current environmental and intrinsic stimuli. This leads to two interesting aspects of eosinophilia: dysbiosis and allergy.

It may be postulated that dysbiosis causes eosinophilia by impairing the integrity of the gut barrier. However, the evidence for this is limited. The presence of *Ruminococcus gnavus* (*Lachnospiraceae*) in infants associated with atopy and its introduction in animal models resulted in exacerbated Th2 immunity [29]. Eosinophilic esophagitis is associated with a kind of dysbiosis that persists despite treatment [30]. The fact of continued colonization with altered microbiota, despite the reduction in inflammation, is especially important because the inflammation itself can shape the microbiota. A specific example is provided by overexpression of IL-13 in mice, which led to changes in gut bacterial communities, such as reduction in *Firmicutes* and *Proteobacteria* [31]. Moreover, in this animal model, overexpression of IL-13 in the airways alone was sufficient to promote changes in the intestinal microbiota, highlighting the systemic consequences of local inflammation. We not only lack information on the relationship between intestinal microbiota and the abundance of eosinophils in the colonic mucosa, but also on how bacteria exposure in early life could prime the gastrointestinal mucosa for greater or lower eosinophil density. The relationships between nutrition, smoking, bacterial metabolism, abundance of aryl hydrocarbon receptor ligands and eosinophilic disorders also warrant further study [27].

The intestinal mucosa is the main site of food allergy reactions, despite the fact that allergens may cross this barrier and reach the bloodstream. Likewise, the involvement of eosinophils in allergic reactions is important, even though other cells also take part. Therefore, eosinophils are critically important in allergy, but do not produce it alone. This is demonstrated by the aforementioned fact that both anti-IgE and anti-IL-5 strategies prove to be useful in the management of asthma. Allergic and eosinophilic asthma may overlap [32], and in such cases a transition from omalizumab (anti-IgE) to mepolizumab (anti-IL-5) might bring additional benefit [33]. In the gut, similar to the lungs, allergy is a complex pathology, where IgE and/or eosinophilia may be involved and do not need to be reciprocally linked [34]. Some functional disorders might be attributable to subtle forms of allergy that attract eosinophils and mast cells to the mucosa, as demonstrated in functional dyspepsia [35] and irritable bowel syndrome [36] independent of IgE. Also, it was postulated in functional dyspepsia that eosinophil recruitment may be reduced by the use of proton pump inhibitors, highlighting unexpected mechanisms of this drug’s activity [37]. In summary, mucosal eosinophils take part in maintaining homeostasis and developing allergic diseases, but the exact mechanisms—even if they are related to the microbiota—remain elusive.

### 4.2. Challenges in the Clinical Interpretation of Colonic Eosinophilia

Many authors emphasize the difficulty of diagnosing eosinophilic colitis due to the frequent lack of coexisting peripheral eosinophilia in this condition [8,9,10,38,39]. Estimates of the occurrence of increased eosinophilic count in the blood in eosinophilic colitis range from 20% to 80% [8,38]. In the diagnostic process, peripheral blood eosinophilia may thus be used to highlight a systemic tendency towards eosinophilia, aiding the diagnostic process, but no strict concordance with histopathology is expected. Of note, very low eosinophil counts were historically considered a sign of ongoing severe inflammation, and therefore the negative predictive value of low eosinophil counts should be considered insufficient. Overall, only high blood eosinophilia appears useful in the diagnosis of eosinophilic diseases of the gut, and its sensitivity is moderate or low. As dictated by clinical experience, a systematic and cautious assessment of the eosinophilic inflammation is required, especially given the considerable overlap with inflammatory bowel diseases already described by Grzybowska-Chlebowczyk et al. [40] This is in addition to the fact that, in some cases, eosinophilic inflammation of the gastrointestinal tract may conclude in fibrosis and mimic surgical emergencies [41].

Studying colonic mucosal eosinophil density in biopsy samples is challenging because eosinophilic infiltration along the colon is frequently patchy [39,42]. A typical example of a condition with patchy lesions in pediatric gastroenterology is celiac disease, where at least five biopsies are required to establish the diagnosis [43]. In eosinophilic esophagitis, multiple specimens are required as well. It is also known that a decreasing gradient of eosinophil density exists along the colon [22,44]. Normal mean values in children range from 20 in the caecum and 16 in the transverse colon to 8 in the sigmoid colon [4]. This was also subject to a meta-analysis that highlighted differences in the real surface of HPF [45]. Although in this study it cannot be ruled out that biopsies, collected during colonoscopy, were taken out of colon segments where eosinophils density was low, the number of mucosal eosinophil density assessments was considerable (~250). This should make the results representative, unless a specific biopsy location bias existed.

### 4.3. Association of Mucosal Eosinophilia with Inflammation, but Not Serum IgE

The limited value of total serum IgE level in diagnosing eosinophilic gastrointestinal diseases was demonstrated previously [46,47]. We nevertheless decided to check for an association between total IgE and mucosal eosinophil density in the colon because of the common functional pathways. Previously, such a relationship was reported in an analysis of 32 children with eosinophilic colitis [22]. Our work points towards a lack of correlation between total serum IgE and colonic mucosal eosinophil density. On the contrary, CRP and WBC correlated with the presence of eosinophils in the colon, stressing the relationship between inflammation in general and eosinophilic infiltration.

### 4.4. Results Reflect Specific Eosinophil Regulation in Younger Children

The lack of a statistically significant relationship between the studied parameters in the younger half of the group can be explained by fundamental differences in immune system regulation. This was studied in eosinophilic esophagitis, where children typically have a background of food allergens, and adults present with sensitization to inhaled allergens [48]. It was shown that molecular profiles of eosinophils differ depending on age [49].

### 4.5. Specific Aspects and Limitations of This Study

As presented above, many of the patients in our study were diagnosed with inflammatory bowel disease, which in itself often causes secondary eosinophilic mucosal infiltration [9,50]. Nevertheless, the underlying condition does not necessarily cause peripheral eosinophilia. Moreover, in many patients, inflammatory bowel disease was suspected, but not confirmed. This study aimed at investigating a general relationship between blood and colon eosinophilia, across age groups, conditions, and various biopsy sites. Although a group selection bias may exist (related to indications for colonoscopy in children), the eosinophil is currently not thought to be the central cell of inflammatory bowel disease or irritable bowel syndrome. Likewise, differentiating between primary and secondary colonic eosinophilia is a current challenge in clinical practice [24]. If a strong general mechanism linking blood and colon eosinophilia existed in children, it should be detectable in the studied population.

Study aspects that warrant consideration are the lack of information on biopsy sites (healthy vs. ulcerated mucosa), endoscopic scores, symptoms and medication. This study also did not include children below 1 year of age. Yet, it managed to explore a number of pertinent parameters across over one hundred patients, describing the strength of the relationship between blood and colonic eosinophilia. This is potentially useful information, given how frequently these parameters are assessed and what uncertainty often surrounds their practical interpretation.

## 5. Conclusions

In conclusion, we observed a modest relationship between blood eosinophil count and the colonic mucosal eosinophil density in symptomatic children who underwent a diagnostic colonoscopy. This association was moderately strong in adolescents and patients with elevated CRP. From a practical standpoint, colonic eosinophilia in children seems to be an unspecific finding that may or may not be accompanied by blood eosinophilia. Colonic mucosal eosinophil density correlated with inflammation (CRP, WBC), but not total serum IgE levels. Investigating healthy adults undergoing colon cancer screening presents itself as an attractive, complementary subject which will facilitate further fundamental study into this topic.

## Figures and Tables

**Table 1:** Main characteristics of patients depending on final diagnosis subgroup.

Characteristic	UC	CD	Other
*n*	34	16	59
Gender—female, %	64.7%	37.5%	40.7%
Age, years	11.7 (8.2–15.5)	13.7 (10.8–16.4)	12.1 (8.0–15.5)
Weight, kg	43.7 (23.4–53.5)	44.0 (27.3–61.1)	39.0 (28.0–62.0)
Height, cm	149.5 (126.0–162.0)	161.5 (144–165.5)	166.9 (131.0–151.0)
CRP, mg/dL	0.13 (0.005–0.99)	1.1 (0.2–2.9)	0 (0–0.08)
WBC, G/L	8.3 (6.3–11.3)	8.5 (7.0–9.4)	6.8 (5.5–8.4)
Eosinophils, G/L	0.18 (0.09–0.40)	0.14 (0.12–0.27)	0.17 (0.10–0.24)
Eosinophils, %	2.2% (1.2–3.9%)	2.2% (1.6–3.4%)	2.6% (1.6–3.5%)
IgE, kU/L	23 (12–87)	81 (56–284)	55 (11–152)
Colonic mucosal eosinophil density, per HPF	32 (25–39)	19 (10–24)	18 (7–24)

CD—Crohn’s disease; CRP—C-reactive protein; HPF—high-power field; UC—ulcerative colitis; WBC—white blood cell.

**Table 2:** Correlations between colonic mucosal eosinophil density and other parameters in all the subjects and in selected subgroups. Spearman’s rho (r) is presented, along with 95% confidence intervals (95% CI). *p* values < 0.05 are indicated with asterisk (*).

**Assessed Correlation, in All Subjects**	**r**	**95% CI**	***p***
colonic mucosal eosinophil density vs. blood eosinophil count	0.295	0.108–0.462	0.0018 *
colonic mucosal eosinophil density vs. blood eosinophil count if CRP < 0.5 mg/dL	0.245	0.022–0.445	0.027 *
colonic mucosal eosinophil density vs. blood eosinophil count if CRP > 0.5 mg/dL	0.529	0.167–0.766	0.0054 *
colonic mucosal eosinophil density vs. relative eosinophil count in the blood	0.175	−0.019–0.356	0.069
colonic mucosal eosinophil density vs. total WBC count	0.262	0.072–0.433	0.0059 *
colonic mucosal eosinophil density vs. CRP	0.182	−0.013–0.364	0.060
colonic mucosal eosinophil density vs. total IgE levels	−0.024	−0.254–0.208	0.833
			* if *p* < 0.05
**Assessed Correlation, in Adolescents > 12.4 Years**	**r**	**95% CI**	***p***
colonic mucosal eosinophil density vs. blood eosinophil count in subjects > 12.4 years	0.448	0.197–0.644	0.00068 *
colonic mucosal eosinophil density vs. relative eosinophil count in the blood in subjects > 12.4 years	0.432	0.177–0.632	0.0011 *
colonic mucosal eosinophil density vs. total WBC count in subjects > 12.4 years	0.300	0.026–0.531	0.028 *
			* if *p* < 0.05

## Data Availability

Data are available from the authors at reasonable request.

## References

[1] Chusid M.J. (2018). Eosinophils: Friends or Foes?. J. Allergy Clin. Immunol. Pract..

[2] Lacy P., Rosenberg H.F., Walsh G.M. (2021). Molecular Biology of Eosinophils: Introduction. Methods Mol. Biol..

[3] Roufosse F., Weller P.F. (2010). Practical approach to the patient with hypereosinophilia. J. Allergy Clin. Immunol..

[4] DeBrosse C.W., Case J.W., Putnam P.E., Collins M.H., Rothenberg M.E. (2006). Quantity and Distribution of Eosinophils in the Gastrointestinal Tract of Children. Pediatr. Dev. Pathol..

[5] Kovalszki A., Weller P.F. (2016). Eosinophilia. Prim. Care.

[6] Huang L., Gebreselassie N.G., Gagliardo L.F., Ruyechan M.C., Lee N.A., Lee J.J., Appleton J.A. (2014). Eosinophil-derived IL-10 supports chronic nematode infection. J. Immunol..

[7] Rauscher C., Freeman A. (2018). Drug-induced eosinophilia. Allergy Asthma Proc..

[8] Impellizzeri G., Marasco G., Eusebi L.H., Salfi N., Bazzoli F., Zagari R.M. (2019). Eosinophilic colitis: A clinical review. Dig. Liver Dis..

[9] Alfadda A.A., Storr M.A., Shaffer E.A. (2011). Eosinophilic colitis: Epidemiology, clinical features, and current management. Ther. Adv. Gastroenterol..

[10] Koutri E., Papadopoulou A. (2018). Eosinophilic Gastrointestinal Diseases in Childhood. Ann. Nutr. Metab..

[11] Papadopoulou A., Koletzko S., Heuschkel R., Dias J.A., Allen K.J., Murch S.H., Chong S., Gottrand F., Husby S., Lionetti P. (2014). Management guidelines of eosinophilic esophagitis in childhood. J. Pediatr. Gastroenterol. Nutr..

[12] Ullmann N., Bossley C.J., Fleming L., Silvestri M., Bush A., Saglani S. (2013). Blood eosinophil counts rarely reflect airway eosinophilia in children with severe asthma. Allergy.

[13] Bedolla-Barajas M., Raúl Ortiz-Peregrina J., Daniel Hernández-Colín D., Morales-Romero J., Ramses Bedolla-Pulido T., Larenas-Linnemann D. (2019). The characterization of asthma with blood eosinophilia in adults in Latin America. J. Asthma.

[14] Zeiger R.S., Schatz M., Li Q., Chen W., Khatry D.B., Gossage D., Tran T.N. (2014). High blood eosinophil count is a risk factor for future asthma exacerbations in adult persistent asthma. J. Allergy Clin. Immunol. Pract..

[15] Baxi S., Gupta S.K., Swigonski N., Fitzgerald J.F. (2006). Clinical presentation of patients with eosinophilic inflammation of the esophagus. Gastrointest. Endosc..

[16] Konikoff M.R., Blanchard C., Kirby C., Buckmeier B.K., Cohen M.B., Heubi J.E., Putnam P.E., Rothenberg M.E. (2006). Potential of Blood Eosinophils, Eosinophil-Derived Neurotoxin, and Eotaxin-3 as Biomarkers of Eosinophilic Esophagitis. Clin. Gastroenterol. Hepatol..

[17] Straumann A., Conus S., Degen L., Felder S., Kummer M., Engel H., Bussmann C., Beglinger C., Schoepfer A., Simon H. (2010). Budesonide Is Effective in Adolescent and Adult Patients with Active Eosinophilic Esophagitis. Gastroenterology.

[18] Rodríguez-Sánchez J., Gómez-Torrijos E., de-la Santa-Belda E., López-Viedma B., Martín-Dávila F., Pilkington-Woll J.P., Donado-Palencia P., Sánchez-Miranda P., Olmedo-Camacho J. (2013). Effectiveness of serological markers of eosinophil activity in monitoring eosinophilic esophagitis. Rev. Esp. Enferm. Dig..

[19] Haasnoot M.L., Mookhoek A., Duijvestein M., D’Haens G.R.A.M., Bredenoord A.J. (2022). Prognostic Value of Colonic Tissue and Blood Eosinophils in Ulcerative Colitis. Inflamm. Bowel. Dis..

[20] Morgenstern S., Brook E., Rinawi F., Shamir R., Assa A. (2017). Tissue and peripheral eosinophilia as predictors for disease outcome in children with ulcerative colitis. Dig. Liver Dis..

[21] Barrie A., Mourabet M.E., Weyant K., Clarke K., Gajendran M., Rivers C., Park S.Y., Hartman D., Saul M., Regueiro M. (2013). Recurrent Blood Eosinophilia in Ulcerative Colitis Is Associated with Severe Disease and Primary Sclerosing Cholangitis. Dig. Dis. Sci..

[22] Behjati S., Zilbauer M., Heuschkel R., Phillips A., Salvestrini C., Torrente F., Bates A.W. (2009). Defining Eosinophilic Colitis in Children: Insights From a Retrospective Case Series. J. Pediatr. Gastroenterol. Nutr..

[23] Martins T.B., Bandhauer M.E., Bunker A.M., Roberts W.L., Hill H.R. (2014). New childhood and adult reference intervals for total IgE. J. Allergy Clin. Immunol..

[24] Walker M.M., Potter M.D., Talley N.J. (2019). Eosinophilic colitis and colonic eosinophilia. Curr. Opin. Gastroenterol..

[25] Smith P.D., Blumberg R.S., Macdonald T.T. (2020). Society for Mucosal Immunology (Menomonee Falls, Wisconsin). Principles of Mucosal Immunology.

[26] Matucci A., Vultaggio A., Maggi E., Kasujee I. (2018). Is IgE or eosinophils the key player in allergic asthma pathogenesis? Are we asking the right question?. Respir. Res..

[27] Diny N.L., Schonfeldova B., Shapiro M., Winder M.L., Varsani-Brown S., Stockinger B. (2022). The aryl hydrocarbon receptor contributes to tissue adaptation of intestinal eosinophils in mice. J. Exp. Med..

[28] Su K.-W., Shreffler W.G., Yuan Q. (2021). Gastrointestinal immunopathology of food protein-induced enterocolitis syndrome and other non-immunoglobulin E-mediated food allergic diseases. Ann. Allergy Asthma Immunol..

[29] Chua H.-H., Chou H.-C., Tung Y.-L., Chiang B.-L., Liao C.-C., Liu H.-H., Ni Y.-H. (2018). Intestinal Dysbiosis Featuring Abundance of Ruminococcus gnavus Associates With Allergic Diseases in Infants. Gastroenterology.

[30] Harris J.K., Fang R., Wagner B.D., Choe H.N., Kelly C.J., Schroeder S., Moore W., Stevens M.J., Yeckes A., Amsden K. (2015). Esophageal microbiome in eosinophilic esophagitis. PLoS ONE.

[31] Sohn K.-H., Baek M.-G., Choi S.-M., Bae B., Kim R.Y., Kim Y.-C., Kim H.-Y., Yi H., Kang H.-R. (2020). Alteration of Lung and Gut Microbiota in IL-13-Transgenic Mice Simulating Chronic Asthma. J. Microbiol. Biotechnol..

[32] Chen M., Shepard K., Yang M., Raut P., Pazwash H., Holweg C.T.J., Choo E. (2021). Overlap of allergic, eosinophilic and type 2 inflammatory subtypes in moderate-to-severe asthma. Clin. Exp. Allergy.

[33] Chapman K.R., Albers F.C., Chipps B., Muñoz X., Devouassoux G., Bergna M., Galkin D., Azmi J., Mouneimne D., Price R.G. (2019). The clinical benefit of mepolizumab replacing omalizumab in uncontrolled severe eosinophilic asthma. Allergy.

[34] Hu Y., Liu S., Liu P., Mu Z., Zhang J. (2020). Clinical relevance of eosinophils, basophils, serum total IgE level, allergen-specific IgE, and clinical features in atopic dermatitis. J. Clin. Lab. Anal..

[35] Cirillo C., Bessissow T., Desmet A.-S., Vanheel H., Tack J., Vanden Berghe P. (2015). Evidence for neuronal and structural changes in submucous ganglia of patients with functional dyspepsia. Am. J. Gastroenterol..

[36] Fritscher-Ravens A., Pflaum T., Mösinger M., Ruchay Z., Röcken C., Milla P.J., Das M., Böttner M., Wedel T., Schuppan D. (2019). Many Patients with Irritable Bowel Syndrome Have Atypical Food Allergies Not Associated with Immunoglobulin E. Gastroenterology.

[37] Wauters L., Ceulemans M., Frings D., Lambaerts M., Accarie A., Toth J., Mols R., Augustijns P., De Hertogh G., Van Oudenhove L. (2021). Proton Pump Inhibitors Reduce Duodenal Eosinophilia, Mast Cells, and Permeability in Patients with Functional Dyspepsia. Gastroenterology.

[38] Uppal V., Kreiger P., Kutsch E. (2016). Eosinophilic Gastroenteritis and Colitis: A Comprehensive Review. Clin Rev Allergy Immunol.

[39] Talley N.J., Shorter R.G., Phillips S.F., Zinsmeister A.R. (1990). Eosinophilic gastroenteritis: A clinicopathological study of patients with disease of the mucosa, muscle layer, and subserosal tissues. Gut.

[40] Grzybowska-Chlebowczyk U., Horowska-Ziaja S., Kajor M., Więcek S., Chlebowczyk W., Woś H. (2017). Eosinophilic colitis in children. Adv. Dermatol. Allergol..

[41] Sheikh R.A., Prindiville T.P., Pecha R.E., Ruebner B.H. (2009). Unusual presentations of eosinophilic gastroenteritis: Case series and review of literature. World J. Gastroenterol..

[42] Ong G.-Y., Hsu C.-C., Changchien C.-S., Lu S.-N., Huang S.-C. (2002). Eosinophilic gastroenteritis involving the distal small intestine and proximal colon. Chang Gung. Med. J..

[43] Husby S., Koletzko S., Korponay-Szabó I., Kurppa K., Mearin M.L., Ribes-Koninckx C., Shamir R., Troncone R., Auricchio R., Castillejo G. (2020). European Society Paediatric Gastroenterology, Hepatology and Nutrition Guidelines for Diagnosing Coeliac Disease 2020. J. Pediatr. Gastroenterol. Nutr..

[44] Bates A.W.H. (2012). Diagnosing Eosinophilic Colitis: Histopathological Pattern or Nosological Entity?. Scientifica.

[45] Kiss Z., Tél B., Farkas N., Garami A., Vincze Á., Bajor J., Sarlós P., Márta K., Eros A., Mikó A. (2018). Eosinophil Counts in the Small Intestine and Colon of Children Without Apparent Gastrointestinal Disease: A Meta-analysis. J. Pediatr. Gastroenterol. Nutr..

[46] Licari A., Votto M., D’Auria E., Castagnoli R., Caimmi S.M.E., Marseglia G.L. (2020). Eosinophilic Gastrointestinal Diseases in Children: A Practical Review. Curr. Pediatr. Rev..

[47] Ko H.M., Morotti R.A., Yershov O., Chehade M. (2014). Eosinophilic Gastritis in Children: Clinicopathological Correlation, Disease Course, and Response to Therapy. Am. J. Gastroenterol..

[48] Straumann A., Aceves S.S., Blanchard C., Collins M.H., Furuta G.T., Hirano I., Schoepfer A.M., Simon D., Simon H.-U. (2012). Pediatric and adult eosinophilic esophagitis: Similarities and differences. Allergy.

[49] Lingblom C., Käppi T., Bergquist H., Bove M., Arkel R., Saalman R., Wennerås C. (2017). Differences in eosinophil molecular profiles between children and adults with eosinophilic esophagitis. Allergy.

[50] Uzunismail H., Hatemi I., Doğusoy G., Akin O. (2006). Dense eosinophilic infiltration of the mucosa preceding ulcerative colitis and mimicking eosinophilic colitis: Report of two cases. Turk. J. Gastroenterol..

